# Predicting Protein-Protein Interactions from Matrix-Based Protein Sequence Using Convolution Neural Network and Feature-Selective Rotation Forest

**DOI:** 10.1038/s41598-019-46369-4

**Published:** 2019-07-08

**Authors:** Lei Wang, Hai-Feng Wang, San-Rong Liu, Xin Yan, Ke-Jian Song

**Affiliations:** 1 College of Information Science and Engineering, Zaozhuang University, Zaozhuang, Shandong 277100 P.R. China; 2 Xinjiang Technical Institute of Physics and Chemistry, Chinese Academy of Sciences, Urumqi, 830011 P.R. China; 3 School of Foreign Languages, Zaozhuang University, Zaozhuang, Shandong 277100 P.R. China; 4 School of information engineering, JiangXi University of Science and Technology, Ganzhou, Jiangxi 341000 P.R. China

**Keywords:** Machine learning, Computational models

## Abstract

Protein is an essential component of the living organism. The prediction of protein-protein interactions (PPIs) has important implications for understanding the behavioral processes of life, preventing diseases, and developing new drugs. Although the development of high-throughput technology makes it possible to identify PPIs in large-scale biological experiments, it restricts the extensive use of experimental methods due to the constraints of time, cost, false positive rate and other conditions. Therefore, there is an urgent need for computational methods as a supplement to experimental methods to predict PPIs rapidly and accurately. In this paper, we propose a novel approach, namely CNN-FSRF, for predicting PPIs based on protein sequence by combining deep learning Convolution Neural Network (CNN) with Feature-Selective Rotation Forest (FSRF). The proposed method firstly converts the protein sequence into the Position-Specific Scoring Matrix (PSSM) containing biological evolution information, then uses CNN to objectively and efficiently extracts the deeply hidden features of the protein, and finally removes the redundant noise information by FSRF and gives the accurate prediction results. When performed on the PPIs datasets *Yeast* and *Helicobacter pylori*, CNN-FSRF achieved a prediction accuracy of 97.75% and 88.96%. To further evaluate the prediction performance, we compared CNN-FSRF with SVM and other existing methods. In addition, we also verified the performance of CNN-FSRF on independent datasets. Excellent experimental results indicate that CNN-FSRF can be used as a useful complement to biological experiments to identify protein interactions.

## Introduction

Protein is the essential component of the living organism, and it participates in various processes of life activities such as metabolism, signal transduction, hormone regulation, DNA transcription and replication^1,2^. In general, proteins perform their functions in the form of complexes by interacting with other proteins. Studying protein-protein interactions (PPIs) not only help to understand the life process, but also help to explore the pathogenesis of disease and pursue drug targets^3^. Over the past several decades, the detection methods of protein interaction based on biological experiments, such as tandem affinity purification (TAP)^4^, yeast two-hybrid (Y2H)^5,6^ and mass spectrometric protein complex identification^7^, gradually matured and achieved considerable research results.

However, due to the biological experiment methods are costly and time-consuming, the protein interaction detected by experimental methods can only account for a small part of the complete PPIs networks^8–11^. In addition, the detection results are also susceptible to the experimental environment and operational processes, resulting in some false positives and false negatives. Therefore, developing reliable computational methods to predict protein interactions accurately is of great practical significance.

In fact, there are many computational methods that have been proposed as complementary to experimental methods to predict protein-protein interactions^12–15^. These methods typically use binary classification model to describe protein-protein pairs with or without interaction, which can be roughly divided into the following categories: protein domains, gene expression, gene neighborhood, protein structure information^16,17^, literature mining knowledge^18^, and phylogenetic profiles^19,20^. However, if there is no corresponding pre-knowledge, these methods cannot be implemented^21,22^.

With the rapid development of sequencing technology, protein sequence information is collected and stored in large quantities. There is abundant useful information in the protein sequence, and the experimental results show that using amino acid sequence alone is sufficient to predict the interaction of protein accurately. Therefore, protein interaction prediction methods that directly extract information from amino acid sequences have aroused great interest in recent years^23–25^. You *et al*. proposed the method of protein interaction prediction based on Support Vector Machine (SVM), considering the sequence order and the dipeptide information of the primary protein sequence. This method has achieved 90.06% accuracy, 94.37% specificity and 85.74% sensitivity in the protein *Yeast* dataset^26^. Hu *et al*. introduced a novel co-evolutionary feature extraction method, namely CoFex, to predict protein interactions. CoFex can extract the feature vectors that accurately express the protein properties according to the presence or absence of the co-evolutionary features of the two protein sequences, thereby providing the performance of the PPIs prediction^27^. Pan *et al*. proposed a new hierarchical LDA-RF model to directly predict protein-protein interactions in the primary protein sequences, which can mine hidden internal structures buried into the noisy amino acid sequences in low-dimensional latent semantic space. The experimental results show that this model can effectively predict potential protein interactions^9^. Saha *et al*. constructed an ensemble model for protein interaction prediction based on a majority voting method. The model uses four well-established machine learning methods: support vector machines, random forests, decision trees, and naive Bayes. In the cross-validation experiment, the ensemble learning method achieved over 80% sensitivity and 90% prediction accuracy^28^. Jeong *et al*. predict protein interactions using algorithms that extract features only from protein sequences and machine learning for computational function prediction. The experimental results show that these features derived from the position-specific scoring matrix are very suitable for protein interaction prediction^29^.

In this study, we propose a novel sequence-based approach, namely CNN-FSRF, to predict potential protein interactions using deep learning Convolutional Neural Network (CNN) algorithm combined with Feature-Selective Rotation Forest (FSRF) classifier. More specifically, we first use the position-specific scoring matrix to convert each protein alphabet sequence into the numerically matrix-based protein descriptor that contains evolution information. Then we use the convolutional neural network to extract the high-level abstraction features of the protein automatically and objectively. Finally, these features are fed into the feature-selective rotation forest classifier to get the final prediction results. To evaluate the predictive performance of CNN-FSRF, we performed verification in the *Yeast* and *Helicobacter pylori* PPI datasets, respectively. The experimental results show that CNN-FSRF achieves 97.75% and 88.96% accuracy with 99.61% and 91.86% sensitivity at the specificity of 95.89% and 86.11% in the above datasets, respectively. Excellent results indicate that CNN-FSRF can be a useful complement to biological experiments to identify potential protein-protein interactions.

## Materials and Methodology

**Figure 1:**
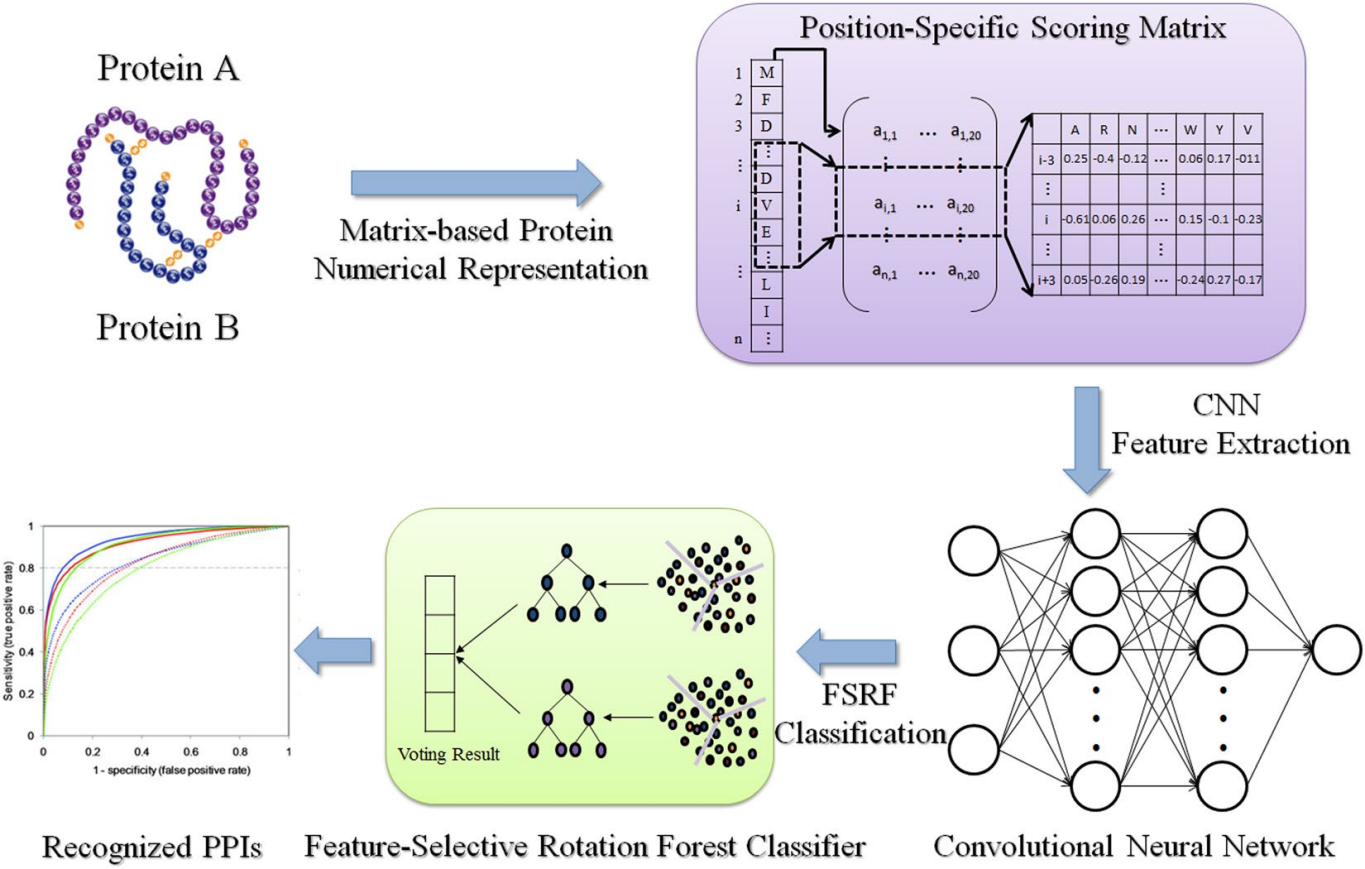
The schematic diagram for predicting protein-protein interactions by integrating convolutional neural network with feature-selective rotation forest model.

In this section, we outline the main idea behind CNN-FSRF approach. Figure 1 gives a schematic diagram of how CNN-FSRF uses convolution neural network and feature-selective rotation forest classifier to predict protein-protein interactions. As can be seen from the figure, our model can be divided into three steps. The first is matrix-based protein numerical representation. For a given protein, since its sequence is usually represented by the letter symbol of 20 kinds of amino acids, in order to facilitate computer algorithm processing, we use the Position-Specific Scoring Matrix (PSSM) method to convert the letter sequence of the protein into the numerical matrix. The second is feature extraction based on Convolutional Neural Network (CNN). Although the protein sequence contains abundant information, it also mixed with a lot of noise. In order to get a more precise representation, we use the deep learning CNN algorithm to extract its features. CNN can automatically and objectively extract the advanced features of protein information in a layer-by-layer manner, thus effectively avoiding the interference of human factors. The finally is the PPI prediction based on Feature-Selective Rotation Forest (FSRF) classifier. After obtaining the advanced features of the protein, we used FSRF classifier to predict relationship between them. The FSRF classifier has the advantage of greatly improving the classification speed under the premise of guaranteeing the accuracy, so as to quickly and effectively predicts the interaction between proteins.

### Golden standard datasets

We evaluate the CNN-FSRF approach through two real PPIs datasets. The *Yeast* dataset collected from the *Saccharomyces cerevisiae* core subset of the Database of Interacting Proteins (DIP) by Guo *et al*.^30^. The core subset contains a total of 5966 interacting protein pairs. After we remove protein pairs containing less than 50 residues or more than 40% sequence identity protein, the remaining 5594 protein pairs constitute the golden standard positive data set. For the standard negative data set, we constructed based on the assumption of Guo *et al*.^30^ that there is no interaction between proteins in different subcellular compartments. To avoid the occurrence of imbalanced dataset, we selected the same number of protein pairs as the positive dataset to construct the negative dataset. As a result, there is a total of 11188 protein pairs in the final *Yeast* dataset, with positive and negative samples each accounting for half. For the *Helicobacter pylori* PPIs dataset from Martin *et al*.^12^, we use the same method for processing. The final *Helicobacter pylori* dataset consisted of 2916 protein pairs, of which 1458 interacted pairs and 1458 non-interacted pairs.

### Evaluation criteria

To evaluate the performance of CNN-FSRF, we use the 5-fold cross-validation and several general evaluation criteria in our experiments. The 5-fold cross-validation randomly divides whole dataset into five independent subsets of the same size. Each time one subset is used as the test set, and the remaining four subsets are used as the training sets. In the experiment, this process is executed five times to ensure that each subset is used as the test set once. Finally, the average and standard deviation of these five experiments are taken as the final experimental results. We follow the widely used evaluation criteria to evaluate the model, including accuracy (Accu.), sensitivity (Sen.), specificity (Spec.), precision (Prec.), F-Score (*F*_*score*_), and Matthews Correlation Coefficient (MCC). They are defined as:

1 $$Accu.=\frac{TP+TN}{TP+TN+FP+FN}$$

2 $$Sen.=\frac{TP}{TP+FN}$$

3 $$Spec.=\frac{TN}{TN+FP}$$

4 $$Prec.=\frac{TP}{TP+FP}$$

5 $${F}_{score}=2\times \frac{Sen.\times Prec.}{Sen.+Prec.}$$

6 $$MCC=\frac{TP\times TN-FP\times FN}{\sqrt{(TP+FP)(TP+FN)(TN+FP)(TN+FN)}}$$

where TP indicates the number of positive samples that are correctly identified, TN indicates the number of negative samples that are correctly identified, FP indicates the number of positive samples that are incorrectly identified, and FN indicates the number of negative samples that are incorrectly identified.

In these evaluation criteria, the accuracy reflects the proportion of the correct prediction results of the model. Sensitivity reflects the ability of classification model to identify positive samples. The higher value of sensitivity indicates that the model has a stronger ability to identify positive samples. Precision reflects the ability of classification model to discriminate negative samples. The higher value of precision indicates that the model has a stronger ability to discriminate negative samples. F_score_ is a combination of sensitivity and precision. The higher value of F_score_ indicates that the model is more robust. The Matthew correlation coefficient (MCC) reflects the correlation between the prediction results and the observation results. It is an important indicator of the overall performance of the model. The larger value of MCC indicates that the model has a better performance. In addition, Receiver Operating Characteristic (ROC) curves and Precision-Recall (P-R) curves are also drawn as evaluation criteria. In order to directly measure the quality of the results expressed by the ROC curve, the Area Under a Curve (AUC) is calculated at the same time. Its value ranges from 0 to 1 and the larger the value, the better the performance of the model.

### Matrix-based protein numerical representation

Protein sequences are usually stored in the database in the form of letters. In order to facilitate the deep learning algorithm to extract its hidden features, the protein sequence must be encoded into the numerical form. In this study, we use the Position-Specific Scoring Matrix (PSSM) method that can contain biological evolution information to generate matrix-based numeric descriptors^31,32^. When measuring the matching weights of amino acids, PSSM not only records the importance and relevance of matching, but also records the position of amino acid residues in the sequence. This matrix helps to reveal more evolutionary information of protein sequences and is therefore widely used in many fields of bioinformatics.

PSSM is the matrix of *N* row of 20 columns, where the row represents the length of the protein sequence and the column represents the 20 naive amino acids. Assume that *P* = {*r*_*i*,*j*_:*i* = 1 … *N and j* = 1 … 20}, PSSM can be expressed as:

7 $$P=[\begin{array}{cccc}{r}_{1,1} & {r}_{1,2} & \cdots  & {r}_{1,20}\\ {r}_{2,1} & {r}_{2,2} & \cdots  & {r}_{2,20}\\ \vdots  & \vdots  & \vdots  & \vdots \\ {r}_{N,1} & {r}_{N,2} & \cdots  & {r}_{N,20}\end{array}]$$

where *r*_*i*,*j*_ in the *i* row of PSSM mean that the probability of the *ith* residue being mutated into type *j* of 20 native amino acids during the procession of evolutionary in the protein from multiple sequence alignments.

In the experiment, we use the sequence comparison tool Position-Specific Iterated BLAST (PSI-BLAST) to obtain the PSSM matrix. BLAST is an effective tool for finding locally similar regions between sequences. It is able to compare nucleotide or protein sequences to sequence databases, and calculate the statistical significance of matches, so as to infer the functional and evolutionary relationships between sequences as well as help identify gene family members. PSI-BLAST is a more sensitive BLAST program that can effectively detect new members of protein families and similar proteins in distantly related species. The feature of PSI-BLAST is that it can use the profile to search the database, re-construct the profile with the results of the search, and then search the database again with the new profile, so repeatedly until no new results are produced. PSI-BLAST naturally extends the BLAST method to find hidden patterns in protein sequences and to find many related proteins with a large sequence difference and a similar structural function. To maximize the effectiveness of the algorithm, we use the non-redundant *SwissProt* as the alignment database. All sequence entries in the *SwissProt* database are searched by experienced protein chemists and molecular biologists for consulting the relevant literature and carefully checking through computer tools. In addition, we also set the expected threshold of the PSI-BLAST algorithm to 0.001, the number of iterations to 3, and the rest of the parameters to the default values.

### Convolutional neural network

Deep learning belongs to a branch of machine learning. Its motivation lies in establishing and simulating the neural network of the human brain for learning, and interpreting data in a mechanism that imitates the human brain^33–35^. Deep learning can form an abstract high-level representation by combining low-level features to discover the rules of data. Therefore, in this paper, we use deep learning convolution neural network algorithm to extract hidden useful information in protein.

**Figure 2:**
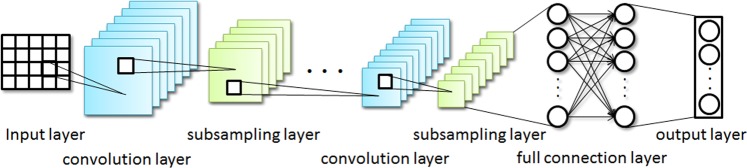
Convolution Neural Network Structure Diagram.

The convolution neural network is a feed-forward neural network. Its neurons can respond to the surrounding units in a part of the coverage and have excellent performance for data feature extraction^36^. CNN uses forward propagation to calculate the output value and back propagation to adjust weights and biases. CNN is composed of the input layer, the convolution layer, subsampling layer, full connection layer and the output layer. Its structure diagram is shown in Fig. 2.

Assuming that *L*_*i*_ represents the feature map of the *ith* layer, it can be described as:

8 $${L}_{i}=h({L}_{i-1}\,\circ \,{W}_{i}+{b}_{i})$$

where *W*_*i*_ means the weight matrix of the convolution kernel of *ith* layer, *b*_*i*_ means the offset vector, *h*(*x*) means the activation function and operator $\circ$ means convolution operations. The subsampling layer usually behind the convolutional layer and the feature map is sampled according to given rules. Assuming that *L*_*i*_ is a subsampling layer, its sampling formula is:

9 $${L}_{i}=subsampling({L}_{i-1})$$

Through multiple convolution and sub sampling operations, CNN classifies the extracted features by the fully connected layer, and the probability distribution ${\mathcal F}$ is obtained based on input. The core mathematical idea of CNN is to map the input matrix *L*_o_ to a new feature representation ${\mathcal F}$ through multi-layer data transformation.

10 $$ {\mathcal F} (i)=Map(C={c}_{i}|{L}_{0};\,(W,b))$$

where *c*_*i*_ represents the *ith* label class, *L*_o_ denotes the input matrix, and ${\mathcal F}$ denotes the feature expression.

The goal of CNN training is to minimize the network loss function *F*(*W*, *b*). At the same time, to alleviate the over fitting problem, the final loss function *E*(*W*, *b*) is usually controlled by a norm, and the intensity of the over fitting is controlled by the parameter ε.

11 $$E(W,b)=F(W,b)+\frac{{\rm{\varepsilon }}}{2}{W}^{T}W$$

When adjusting parameters, CNN usually uses gradient descent method to optimize, update network parameters (*W*, *b*) layer by layer from back to front, and use learning rate *λ* to control the strength of back propagation.

12 $${W}_{i}={W}_{i}-{\rm{\lambda }}\frac{\partial E(W,b)}{\partial {W}_{i}}$$

13 $${b}_{i}={b}_{i}-{\rm{\lambda }}\frac{\partial E(W,b)}{\partial {b}_{i}}$$

### Feature-selective rotation forest

The Rotation Forest (RF) is an ensemble classifier which contains multiple decision trees. It can quickly be applied to many data science problems and can efficiently obtain accurate classification results^37^. Therefore, it has received high attention and popularity from researchers. The main idea of RF is to randomly divide the data set into multiple subsets and implement the corresponding coordinate transformation, and transform the data from the original space to the new space to increase the difference between the data, so as to improve the diversity and accuracy of the classifier at the same time.

In this study, aiming at the high dimensionality and noise-containing characteristics of the PPIs data, we improved the RF and proposed Feature-Selective Rotation Forest (FSRF) algorithm. The FSRF algorithm can effectively reduce the data dimension and remove the noise information in the data, thus improving the prediction accuracy and speed of the classifier. More specifically, we use the *χ*^2^ method in statistics to calculate the weight of all the features, and rank them according to the weighted values, and delete the small influence on the classification according to the given feature selection rate. The weight of a given feature *P* can be calculated according to the following formula.

14 $${\chi }^{2}=\sum _{i=1}^{l}\sum _{j=1}^{2}\frac{{({\rho }_{ij}-{\sigma }_{ij})}^{2}}{{\sigma }_{i,j}}$$

where *l* is the number of values in feature *P*, *ρ*_ij_ is the count of the value *β*_*i*_ in feature *P* belongs to class *y*_*j*_, defined as:

15 $${\rho }_{ij}=count(P={\beta }_{i}\,and\,Y={y}_{j})$$

*σ*_*i*,*j*_ is the expected value of *β*_*i*_ and *y*_*j*_, defined as:

16 $${\sigma }_{i,j}=\frac{count(P={\beta }_{i})\times count(Y={y}_{j})}{L}$$

where *count*(*P* = *β*_*i*_) is the number of samples with the value *β*_*i*_ in the feature *P*, *count*(*Y* = *y*_*j*_) is the number of samples with the value *y*_*j*_ in the class *Y*, and *L* is the total number of samples in the training set.

After calculating the weights of all the features by formula 14, we remove the features with small weight value according to the given weight selection rate *ε*, and thus obtain a new feature set *S*. Let *E* = (*e*_1_, *e*_2_, …, *e*_*n*_)^*T*^ be an *n × L* matrix which is composed of *n* observation feature vector for each training sample and *C* = (*c*_1_, *c*_2_, …, *c*_*n*_)^*T*^ denote the corresponding labels. Therefore, the data sample can be represented as {*e*_*i*_, *c*_*i*_}, where *e*_*i*_ = (*e*_*i*1_, *e*_*i*2_, …, *e*_*iL*_) is an L-dimensional feature vector. According to the number *K* of given decision trees, the sample set is randomly divided into a subset of the same size and transformed by principal component analysis (PCA) algorithm. Then all coefficients of the principal component are rearranged and stored to form a rotation matrix to change the original training set. Therefore, the decision tree can be represented by *T*_1_, *T*_2_, …, *T*_*k*_, and the training process of one decision tree *T*_*i*_ can be described as follows:

- The sample set *S* is randomly divided into *K* (a factor of n) disjoint subsets, and each subset contains the number of features is *n*/*k*.
- A corresponding column of features in the subset *S*_*i*,*j*_ is selected to form a new matrix *E*_*i*,*j*_ from the training dataset *E*. A new training set ${E^{\prime} }_{i,j}$ which is extracted from *E*_*i*,*j*_ randomly with 3/4 of the dataset using bootstrap algorithm. Loop *K* times in this way, so that each subset is converted
- Matrix ${E^{\prime} }_{i,j}$ is used as the feature transform by PCA technique for producing the coefficient matrix *M*_*i*,*j*_, which *j*th column coefficient as the characteristic component *j*th.
- A sparse rotation matrix *R*_*i*_ is constructed, and its coefficients which obtained from the matrix *M*_*i*,*j*_ expressed as follows:

17 $${R}_{i}=[\begin{array}{llll}{\mu }_{i,1}^{(1)},\,\ldots ,\,{\mu }_{i,1}^{({G}_{1})} & 0 & \cdots  & 0\\ 0 & {\mu }_{i,2}^{(1)},\,\cdots ,\,{\mu }_{i,2}^{({G}_{2})} & \cdots  & 0\\ \vdots  & \vdots  & \ddots  & \vdots \\ 0 & 0 & \cdots  & {\mu }_{i,k}^{(1)},\ldots ,{\mu }_{i,k}^{({G}_{k})}\end{array}]$$

In the prediction period, provided the test sample *e*, generated by the classifier *T*_*i*_ of ${d}_{i,j}(E{R}_{i}^{\mu })$ to determine *e* belongs to class *c*_*i*_. And then the class of confidence is calculated by means of the average combination, and the formula is as follows:

18 $${\theta }_{j}(e)=\frac{1}{k}\sum _{i=1}^{k}{d}_{i,j}(E{R}_{i}^{\mu })$$

Therefore, the test sample *e* easily assigned to the classes with the greatest possible.

## Results and Discussion

In this section, we summarize the experimental results of the CNN-FSRF method on the standard datasets. To comprehensively evaluate the performance of the model, we compare the proposed method with the state-of-the-art Support Vector Machine (SVM) classifier and other excellent methods on the same datasets. In addition, we verified the proposed method on independent datasets. The CNN-FSRF based on protein sequence is implemented by MATLAB platform. For the SVM classifier, we use the LIBSVM implementation designed by Lin *et al*., which can be downloaded at https://www.csie.ntu.edu.tw/~cjlin/libsvm/. The parameters of FSRF and SVM algorithms have been optimized by the grid search method.

### Prediction Performance of CNN-FSRF Model

**Figure 3:**
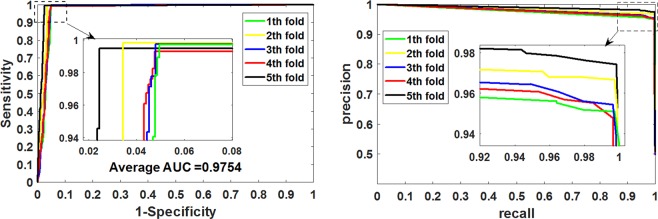
The ROC and P-R curves were generated on the *Yeast* dataset by using the CNN-FSRF method.

We first performed experiments on *Yeast* dataset, and Table 1 summarizes the results of the 5-fold cross-validation experiment. It can be seen that the accuracy of CNN-FSRF on *Yeast* dataset was as high as 97.75%. In order to better investigate the predictive ability of the model, we also calculate the values of sensitivity, specificity, precision, F_score_, Matthews correlation coefficient, and AUC. In these evaluation criteria, the F_score_ value that reflects the stability of the model is 97.79% and the MCC and AUC values that reflect the overall performance of the model were 95.57% and 97.54%, and their standard variance were 0.53%, 1.05% and 0.66%, respectively. Figure 3 shows the ROC curves and P-R curves obtained by CNN-FSRF on the *Yeast* dataset respectively. It can be seen from the graph that the curves generated by the five experiments cover most of the coordinate space. The 5-fold cross-validation experimental results demonstrate that CNN-FSRF performs well on the *Yeast* dataset.

**Table 1:** The 5-fold cross-validation results were generated on the *Yeast* dataset by using the CNN-FSRF method.

Test set	Accu.(%)	Sen.(%)	Spec. (%)	Prec.(%)	Fscore(%)	MCC(%)	AUC(%)
1	97.36	99.73	95.04	95.18	97.40	94.83	96.97
2	98.17	99.82	96.55	96.59	98.18	96.39	97.92
3	97.45	99.73	95.19	95.36	97.50	95.00	97.17
4	97.27	99.29	95.22	95.48	97.35	94.62	97.13
5	98.48	99.47	97.46	97.58	98.52	96.98	98.52
Average	**97**.**75**	**99**.**61**	**95**.**89**	**96**.**04**	**97**.**79**	**95**.**57**	**97**.**54**
Standard Deviation	**0**.**54**	**0**.**22**	**1**.**07**	**1**.**02**	**0**.**53**	**1**.**05**	**0**.**66**

**Figure 4:**
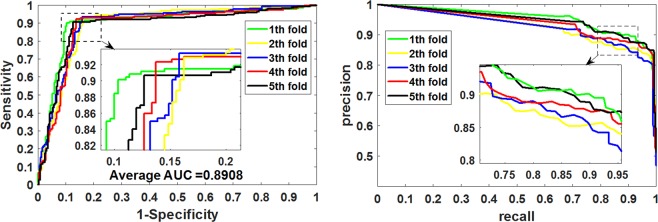
The ROC and P-R curves were generated on the *Helicobacter pylori* dataset by using the CNN-FSRF method.

We next implement the proposed method on the *Helicobacter pylori* dataset, and its 5-fold cross-validation experimental results are shown in Table 2. We can see from Table 2 that CNN-FSRF achieved an accuracy of 88.96% on the *Helicobacter pylori* dataset. In the F_score_, MCC, and AUC that comprehensively reflect model performance, the values obtained by CNN-FSRF were 89.26%, 78.09%, and 89.08%, and the standard deviations were 0.67%, 1.16%, and 0.79%, respectively. Figure 4 plots the ROC curves and P-R curves generated on the *Helicobacter pylori* dataset. It can be seen from the figure that although the CNN-FSRF performance on the *Helicobacter pylori* dataset is not excellent on the *Yeast* dataset, it also achieved good performance. This may be due to the fact that the number of samples in the *Helicobacter pylori* dataset (2916) is less than in the *Yeast* dataset (11188). It is well known that the number of samples used to train the classifier in machine learning is closely related to the final test result. The more samples in the training set, the more fully trained the classifier, the higher the model fitting degree learned, and the better the prediction result. Therefore, the results obtained by the proposed model in the *Helicobacter pylori* dataset were not as good as those in the *Yeast* dataset also conform to this rule. In addition, this result can also indicate that the performance of CNN-FSRF will become better as the training set increases.

**Table 2:** The 5-fold cross-validation results were generated on the *Helicobacter pylori* dataset by using the CNN-FSRF method.

Test set	Accu.(%)	Sen.(%)	Spec. (%)	Prec.(%)	Fscore(%)	MCC(%)	AUC(%)
1	89.88	90.10	89.66	89.80	89.95	79.76	90.08
2	88.34	92.83	83.79	85.27	88.89	76.97	89.24
3	88.51	93.41	84.19	83.88	88.39	77.52	89.54
4	89.37	92.31	86.27	87.62	89.90	78.81	88.21
5	88.70	90.67	86.62	87.74	89.18	77.40	88.35
Average	**88**.**96**	**91**.**86**	**86**.**11**	**86**.**86**	**89**.**26**	**78**.**09**	**89**.**08**
Standard Deviation	**0**.**65**	**1**.**42**	**2**.**34**	**2**.**31**	**0**.**67**	**1**.**16**	**0**.**79**

### Comparison between the proposed model and SVM Model

SVM is a supervised learning model, which is one of the most robust and accurate methods in data mining algorithms^38^. SVM can map the sample space into the high-dimensional feature space through a non-linear mapping, so that the non-linear separable problem in the original sample space is transformed into a linear separable problem in the feature space. To demonstrate the performance of the proposed method, we compare the CNN-FSRF and SVM model (CNN-SVM) on the same dataset. For fairness, we optimized the parameters of the SVM using the grid search method and used the same protein number descriptors.

The 5-fold cross-validation experimental results by the SVM classifier combined with the CNN extracted feature descriptors were shown in Table 3. It is observed from Table 3 that CNN-SVM achieved the 5-fold cross-validation accuracy of 88.92% and the standard deviation of 1.34% on the *Yeast* dataset. The accuracy is 8.83% lower than that of CNN-FSRF and the standard deviation is 0.80% higher than that of CNN-FSRF. Except that CNN-SVM is 0.11% higher than CNN-FSRF on sensitivity, CNN-SVM is 17.76%, 14.01%, 7.79%, 15.84% and 8.69% lower on specificity, precision, F_score_, MCC and AUC than CNN-FSRF. However, in the standard deviation, the above evaluation criteria CNN-SVM are 0.03%, 1.64%, 0.95%, 0.59%, 1.17% and 0.63% higher than CNN-FSRF, respectively.

**Table 3:** Comparison of 5-fold cross-validation results of CNN-FSRF and CNN-SVM on *Yeast* dataset.

Test set	Accu.(%)	Sen.(%)	Spec. (%)	Prec.(%)	Fscore(%)	MCC(%)	AUC(%)
1	89.27	99.73	78.99	82.35	90.21	80.35	88.34
2	87.89	99.91	76.13	80.36	89.08	78.11	88.42
3	89.05	100.00	78.18	81.97	90.09	80.06	89.11
4	87.48	99.56	75.20	80.33	88.92	77.21	87.48
5	90.89	99.38	82.14	85.15	91.72	82.94	90.91
CNN-SVM Average	88.92	**99**.**72**	78.13	82.03	90.00	79.73	88.85
CNN-SVM Standard Deviation	1.34	0.25	2.71	1.97	1.12	2.22	1.29
CNN-FSRF Average	**97**.**75**	99.61	**95**.**89**	**96**.**04**	**97**.**79**	**95**.**57**	**97**.**54**
CNN-FSRF Standard Deviation	**0**.**54**	**0**.**22**	**1**.**07**	**1**.**02**	**0**.**53**	**1**.**05**	**0**.**66**

**Figure 5:**
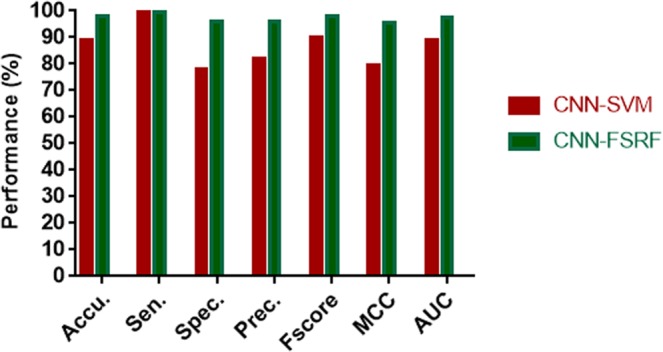
Comparison of performance between CNN-FSRF and CNN-SVM on the *Yeast* dataset.

**Figure 6:**
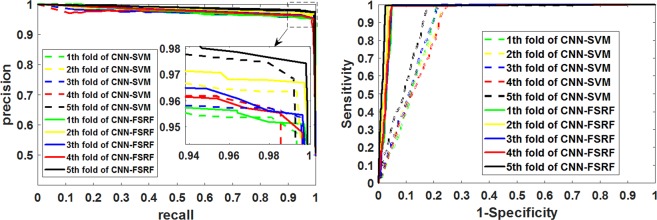
Comparison of ROC curves and P-R curves of CNN-FSRF and CNN-SVM on the same coordinate axis.

To facilitate observation, we present these evaluation criteria in the form of histogram. At the same time, we also plotted ROC curves and P-R curves of CNN-FSRF and CNN-SVM on the same coordinate axis. It can be clearly seen from Fig. 5 that CNN-FSRF performed better than CNN-SVM on accuracy and F_score_, which reflects the prediction accuracy and the stability of the model. In addition, it can be clearly seen from Fig. 6 that the proposed CNN-FSRF also outperforms CNN-SVM on comprehensive evaluation criteria AUC reflecting the overall performance of the model. This indicates that the overall performance of CNN-FSRF is superior to that of CNN-SVM. Therefore, we have reason to believe that the proposed CNN-FSRF method can effectively predict the interaction between proteins.

### Comparison with existing methods

To further evaluate the performance of CNN-FSRF, we collected the work of other researchers on the same *Yeast* and *Helicobacter pylori* datasets and used 5-fold cross-validation method to predict PPI. Since some works do not provide more evaluation criteria, we only list the common evaluation criteria of these works, including accuracy, sensitivity, precision and MCC.

Table 4 lists the performance of several previous works and our model on the *Yeast* dataset. From the table we can see that the proposed method achieves the best results in accuracy, sensitivity and MCC, but only the third result in precision. Specifically, the proposed model achieved 97.75% on the accuracy, which is 1.15% higher than the second highest Wangs’ work. The model has a great advantage in sensitivity, and achieves 99.61% of the results, which is 4.49% higher than the second highest Zhangs’ work. The results obtained from the proposed model on precision generally achieved only the third highest 95.89% result, which was 3.47% lower than the first high Wangs’ work. The proposed model on the MCC also has a large advantage, achieving 96.04% of the results, which is 2.63% higher than the second highest Wangs’ work. Generally speaking, the comprehensive performance of the proposed method is superior to other methods in the table, and has highly competitive in predicting PPI. In addition, we can also see that Wangs’ work, Dus’ work, Zhangs’ work, Patels’ work and the proposed model all use deep learning-based algorithms, and the results obtained by these methods are significantly better than those of other methods in the table that do not use deep learning. This demonstrates that the use of deep learning algorithm can effectively improve the performance of the model.

**Table 4:** The performance comparison between different methods on the *Yeast* dataset.

Author	Model	Accu.(%)	Sen.(%)	Prec.(%)	MCC(%)
Yangs’ work^40^	Cod1	75.08 ± 1.13	75.81 ± 1.20	74.75 ± 1.23	N/A
	Cod2	80.04 ± 1.06	76.77 ± 0.69	82.17 ± 1.35	N/A
	Cod3	80.41 ± 0.47	78.14 ± 0.90	81.86 ± 0.99	N/A
	Cod4	86.15 ± 1.17	81.03 ± 1.74	90.24 ± 0.45	N/A
Zhous’ work^41^	SVM + LD	88.56 ± 0.33	87.37 ± 0.22	89.50 ± 0.60	77.15 ± 0.68
Yous’ work^42^	PCA-EELM	87.00 ± 0.29	86.15 ± 0.43	87.59 ± 0.32	77.36 ± 0.44
Guos’ work^30^	ACC	89.33 ± 2.67	89.93 ± 3.68	88.87 ± 6.16	N/A
	AC	87.36 ± 1.38	87.30 ± 4.68	87.82 ± 4.33	N/A
Wangs’ work^43^	SAE	96.60 ± 0.22	93.73 ± 0.46	**99**.**36** ± **0**.**41**	93.41 ± 0.41
Dus’ work^44^	DeepPPI	94.43 ± 0.30	N/A	96.65 ± 0.59	88.97 ± 0.62
Zhangs’ work^45^	EnsDNN	95.29 ± 0.43	95.12 ± 0.45	95.45 ± 0.89	90.59 ± 0.86
Patels’ work^46^	DeepInteract	92.67	86.85	98.31	85.96
Our model	CNN-FSRF	**97**.**75** ± **0**.**54**	**99**.**61** ± **0**.**22**	95.89 ± 1.02	**96**.**04** ± **1**.**05**

We collected previous work on the *Helicobacter pylori* dataset and summarized the results in Table 5. We can see from the table that our model achieved the best results in terms of accuracy, sensitivity, and precision, and achieved the second best result on the MCC. Specifically, CNN-FSRF is 1.46% higher in accuracy than the second Ensemble ELM model, 2.91% higher in sensitivity than the second Ensemble ELM model, 0.71% higher in precision than the second Ensemble ELM model, and 0.04% lower in MCC than the first Ensemble ELM model. Generally, our model achieved the highest prediction accuracy on the *Helicobacter pylori* dataset, and the performance of the model ranked second, but it is only 0.04% less than the first one.

**Table 5:** The performance comparison of different methods on the *Helicobacter pylori* dataset.

Model	Accu.(%)	Sen.(%)	Prec.(%)	MCC(%)
HKNN	84.00	86.00	84.00	N/A
Boosting^47^	79.52	80.37	81.69	70.64
Signature products^12^	83.40	79.90	85.70	N/A
Ensemble of HKNN^48^	86.60	86.70	85.00	N/A
Ensemble ELM^42^	87.50	88.95	86.15	**78**.**13**
Phylogentic bootstrap^49^	75.80	69.80	80.20	N/A
Our model	**88**.**96**	**91**.**86**	**86**.**86**	78.09

We can also see from Tables 4 and 5 that the performance of these methods we collected on the *Helicobacter pylori* dataset is generally not as good as that on *Yeast* dataset, which is likely to be related to the number of dataset samples, and also in accordance with the conclusions of our previous section. In addition, it can be seen from the horizontal comparison that the results obtained by our model on the *Helicobacter pylori* dataset are only slightly better than the other methods, but the results obtained on the *Yeast* dataset are much better than the other methods. This indicates that with the increase of data sets, our approach can quickly improve overall performance and is well-suited for large datasets.

### Performance on independent datasets

Although CNN-FSRF achieved high light performance on the *Yeast* and *Helicobacter pylori* datasets, we further verify its performance on independent datasets. Specifically, we first train the CNN-FSRF using the entire *Yeast* dataset, and then use the trained model to predict the interaction among the proteins in the *C*. *elegans*, *E*. *coli*, *H*. *sapiens* and *M*. *musculus* datasets. This in biological experiments means using protein interactions identified in one organism to predict interactions in other organisms. This approach is based on the assumption that homologous proteins have the ability to maintain their interactions. The hypothesis is based on the assumption that homologous species have similar functional behaviors, so that they maintain the same PPIs^39^.

The *C*. *elegans*, *E*. *coli*, *H*. *sapiens* and *M*. *musculus* datasets contain only pairs of interacting proteins, the numbers of which are 4013, 6954, 1412, and 313, respectively. Therefore, in the experiment we only calculated meaningful accuracy, sensitivity and F_score_. Table 6 lists the experimental results on the independent datasets. As can be seen from the table, CNN-FSRF achieved good results in these four datasets, with average accuracy, sensitivity, and F_score_ of 95.95%, 95.95% and 97.92%, respectively. Excellent experimental results show that our model can also achieve good results in independent datasets. This fully demonstrates that our method not only has good performance, but also has good generalization and can be applied to different protein interaction prediction problems.

**Table 6:** Prediction results of four species based on the proposed method.

Species	Test pairs	Accu.(%)	Sen.(%)	F_score_(%)
*C*. *elegans*	4013	96.41	96.41	98.17
*E*. *coli*	6954	95.47	95.47	97.68
*H*. *sapiens*	1412	98.65	98.65	99.32
*M*. *musculus*	313	93.27	93.27	96.52

## Conclusions

In this study, we develop a novel sequence-based approach to accurately predict potential protein-protein interactions by combining deep learning convolutional neural network with feature-selective rotation forest. It is well known that extracting effective feature descriptors is the key to predicting PPIs, so the main advantage of this paper is that it can extract the feature information of protein objectively and profoundly by the convolution neural network. Then use FSRF to remove noise information and give accurate prediction results. The experimental results show that CNN-FSRF performs significantly well in predicting PPIs. CNN-FSRF obtained 97.75% and 88.96% prediction accuracy using the 5-fold cross-validation in the real PPIs datasets *Yeast* and *Helicobacter pylori*. In the experiment, we compared the CNN-FSRF with the SVM model and other existing methods. In addition, we validated our approach on the independent datasets. Excellent experimental results demonstrate that our approach can be an effective tool to accurately predict potential protein interactions. In future research, we will continue to study the use of deep learning to extract effective protein features in the hope of achieving better results.
